# Registered nurses’ views on consideration of patient perspectives during multidisciplinary team meetings in cancer care

**DOI:** 10.1186/s12912-022-01127-2

**Published:** 2022-12-09

**Authors:** Linn Rosell, Wenche Melander, Berit Lindahl, Mef Nilbert, Marlene Malmström

**Affiliations:** 1 Regional Cancer Centre South, Region Skåne, Lund, Sweden; 2 Division of Oncology, Department of Clinical Sciences Lund, Lund University, Scheeletorget 1, 22 363 Lund, Sweden; 3 Department of Health Sciences, Faculty of Medicine, Lund University, Lund, Sweden; 4 Department of Surgery and Gastroenterology, Skåne University Hospital, Lund, Sweden; 5 Institute for Palliative Care, Lund University and Region Skåne, Lund, Sweden

**Keywords:** Nurse navigator, Patient perspective, Inter-disciplinary team, Tumour board, Cancer care

## Abstract

**Background:**

Multidisciplinary team meetings (MDTMs) represent an integral component of modern cancer care and have increasingly been implemented to ensure accurate and evidence-based treatment recommendations. During MDTMs, multiple and complex medical and patient-related information should be considered by a multi-professional team whose members contribute various perspectives. Registered nurses (RNs) are expected to share information on the patient perspective at MDTMs. However, research suggests that RNs’ contributions to case discussions are limited and that patient perspective is generally underrepresented. Our aim was to explore RNs’ views of the prerequisites for and barriers to the inclusion of the patient perspective in MDTMs in Swedish cancer care.

**Methods:**

Data were collected from four focus group interviews with 22 RNs who worked as contact nurses in Swedish cancer care. Interviews were transcribed and analysed using inductive content analysis.

**Results:**

The analysis identified two categories and five subcategories. The participants presented different views and expressed ambivalence about the patient perspective in MDTMs. Subcategories were related to medical versus holistic perspectives, the added value of patient perspective, and possibilities for patient contributions. The participants also discussed prerequisites for the patient perspective to be considered in MDTM decision-making process, with subcategories related to structures promoting attention to the patient perspective and determinants of RNs’ contributions to case discussions in MDTMs.

**Conclusions:**

This study demonstrates various views related to the patient perspective in MDTMs and identifies a great need to clarify the RN’s role. Our results indicate that if enhanced presentation of the patient perspective in MDTMs is desired, key information points and structures must be established to collect and present relevant patient-related information.

## Background

In cancer care, treatment recommendations based on multidisciplinary team meetings (MDTMs) represent a key point in the clinical trajectory and aim to ensure diagnostic accuracy and evidence-based treatment recommendations according to best practices and national guidelines [1–3]. The multidisciplinary team (MDT) consists of various professions and disciplines that contribute their expert skills; it generally includes surgeons, oncologists, a pathologist, a radiologist, registered nurses (RNs), and an MDT coordinator [4, 5]. During MDTMs, multiple and complex medical and patient-related information must be considered within a short time frame. Consideration of the patient perspective is suggested to be important in order to provide treatment recommendations that have a high likelihood of being accepted and successfully implemented [4, 6]. RNs are often thought to share information on the patient perspective in MDTMs, but several studies report limited RN contributions to MDTM case discussions and a weak focus on the patient perspective in MDTMs [4, 6–8].

In the context of MDTMs, consideration of information concerning the patient perspective in clinical decision-making is multifaceted [9]. The patient perspective can include information about non-medical characteristics such as age, psychological aspects, and social factors such as family relations, profession, and preferences [10], but can also include medical information on comorbidity and physical status [11]. Several studies report that in MDTMs the biomedical perspective dominates, and less attention is given to the patient perspective [4, 6, 7, 12–14]. This can partly be explained by a lack of strategies to collect this information before MDTMs [14]. However, limited information on the patient perspective in MDTMs may constitute a barrier to individualized treatment recommendations [13, 15, 16], resulting in difficulties implementing the recommendation after the MDTM [9, 11, 17, 18]. At the same time, it is unclear what benefits patients the most: their perspective being included in the decision-making process, or the MDT conducting a fact-based discussion before considering patient preferences [9]. The few studies that have investigated patients’ experiences of involvement in the MDTM decision-making process identify variable patient involvement and preferences, ranging from physician-led decision-making to active patient involvement [19, 20]. Although there are MDTM settings that include patients [17, 21], these are rare. Diekmann et al. [21] reported varied patient experiences of MDTM participation, including both positive and negative experiences, while Lamb et al. [19] described how patients found MDTM participation intimidating, suggesting that RNs should present their perspective. As patients do not generally participate in MDTMs in Sweden, and since there is no standardized structure to collect information on the patient perspective, the term “patient perspective” is used in this study to refer to a holistic view of the unique person, including all relevant aspects that can affect the MDTM discussion and recommendations. Relevant aspects concern patient preferences, care needs, physical status, etc., and this information could be shared by the patient with the responsible physician or RN before the MDTM or be collected from medical records.

As part of the Swedish Cancer Strategy, contact nurses (CNs) were introduced in cancer care 10 years ago. The CN’s role is similar to that of the nurse navigator [22]. CNs are RNs specifically assigned to be the patient’s primary point of contact through a specific cancer trajectory. RNs (i.e., CNs in Sweden) are expected to participate in MDTMs, share information on the patient perspective, and advocate for patients’ interests in the meeting [4, 6, 8, 9, 14, 22, 23]. In this study, we refer to the CN as an RN. As a key member of the MDT, the RN is expected to contribute to the case discussion; however, previous studies have shown limited contributions from RNs compared to other MDTM participants [4, 6–8]. Several reasons for this have been suggested, such as dominance of the medical perspective [24], resource constraints [25], and the fact that MDTM discussions are conducted at an early stage in the clinical trajectory when the RN may not have met with the patient [8]. Another possible reason may be that most communication traditionally occurs between physicians, with limited involvement of RNs in the decision-making process [24]. To gain insight into RNs’ perceptions of the patient perspective, we explored RNs’ views of the prerequisites for and barriers to the inclusion of the patient perspective in MDTMs in Swedish cancer care.

## Methods

This study was conducted as a descriptive, qualitative study with an explorative design. Data were collected from focus group interviews [26]. The content was analysed using inductive content analysis [27] with the aim of describing RNs’ views of the patient perspective during the MDTMs. Reporting was conducted according to the Standards for Reporting Qualitative Research (SRQR) guidelines [28].

### Context

The MDTM is well established in Swedish cancer care, with meetings typically held on a weekly basis in local, regional, and national settings and with frequent use of video links between regional hospitals. The patient’s case is typically discussed at the time of primary diagnosis, but could also be discussed to ensure the best possible treatment in case of recurrence [29]. The MDT composition may vary somewhat depending on the diagnosis and hospital [2, 3], and the chair is most often a physician [30]. Generally, the chair presents the case history and clinical problem, followed by MDT members contributing with their respective information and diagnostic- and treatment-related perspectives. During the case discussion, relevant treatment options are considered, resulting in treatment recommendations or potentially the need for further investigation. After the MDTM, the recommendations are communicated to the patient and, if desired, their next of kin by a physician, often together with the RN, and the final treatment decision is made in collaboration with the patient.

RNs in Sweden have a bachelor’s degree in nursing, and CNs are RNs with a specific assignment. Several higher education institutions in Sweden offer post-graduate education in palliative and oncological care as well as a specialist CN course. However, specialist education is not a requirement to work as a CN. A national mission statement for CNs in Swedish cancer care was developed in 2011 [31]. It states that CNs should participate in MDTMs, but does not specify their role or responsibility in those meetings. The assignment also specifies that CNs should: be accessible to patients; be responsible for providing information, assessing patient needs and offering support; enhance coordination; and ensure patient participation throughout the clinical trajectory [32]. In cancer care, CNs can work in medical as well as surgical departments, although the prerequisites for carrying out the assignment vary, as does the number of patient contacts.

### Participants

Inclusion criteria for the study were being an RN working as a CN and having knowledge and/or experience of MDTMs. Permission to invite the RNs was received from the head of department, who also administered contact information for all RNs at the clinic in question. The participants received an invitation to participate by e-mail, including information about the study and a consent form. To ensure variation in the type of hospital (i.e., university or county hospital), speciality (i.e., medical or surgical), and clinical trajectory (i.e., breast cancer, gynaecological cancer, head and neck cancer, gastrointestinal cancer, urological cancer, and lung cancer), purposive sampling was conducted. In total 22 RNs from one university hospital and one county hospital in southern Sweden participated.

### Data collection

Four focus group interviews were conducted between May and December 2018, three in the university hospital and one in the county hospital. The groups consisted of four to seven participants. All interviews took place in a remote room in the hospital and lasted between 95 minutes and 109 minutes. Before participation in the focus group interviews, participants completed an informed consent form, which was returned by post, by e-mail, or in person. A questionnaire about basic demographic information was completed before the interviews started (Table 1). The researchers followed a semi-structured interview guide that included open-ended questions exploring participants’ views [26]. Examples of overarching questions are: “How is the patient perspective included in MDTMs today?”; “If information on the patient perspective is included, what impact does it have?”; “What patient-related information do you think is important to include in the MDTM discussion and treatment recommendations?“; “Please describe potential barriers to and opportunities for including the patient perspective”; “Are there any potential risks related to not including the patient perspective?”; and “Do the RNs have any responsibilities related to including the patient perspective in the MDTMs?”. When required, we asked probing questions such as “Can you tell us more about that?” to explore the participants’ reasoning. The interview guide was developed by the research team based on experience of clinical practice and on literature in the research field. All focus group interviews were carried out by two researchers (LR and MM) who have training and expertise in qualitative methodologies and a good understanding of MDTMs in cancer care. The researchers took turns as moderator and assistant moderator. The moderator led the discussion, and the assistant moderator took notes and, at the end of each focus group interview, provided a brief summary of the session, on which the participants were encouraged to verify and reflect up on [26]. The interviews were recorded digitally and transcribed verbatim.

**Table 1:** Participants’ demographic data

**Age (years)**	**Number of participants**
36–45	4
46–55	9
≥56	9
**Work experience in cancer care (years)**	
≤9	3
10–20	10
≥21	9
**Participating in MDTMs (meetings per month)**	
≤3	10
4–7	9
≥8	3

### Data analysis

A qualitative content analysis [27] with an inductive approach was conducted, a method chosen because of the limited research on RNs’ perspectives of the MDTMs. The focus group interviews were analysed by three researchers (LR, WM and MM) to obtain broad and complementary analytical perspectives. The researchers independently read the verbatim transcripts several times to get a sense of the whole. Open coding was conducted during reading and the codes were grouped into subcategories. In the abstraction process that followed, the subcategories were grouped under categories based on similarities, and categories were grouped under main categories [27]. The definition of the categories was a dynamic analytical process moving back and forth between the specific and general perspectives, and the categories were developed through discussion between all the authors. The notes were used to increase the trustworthiness of the analysis. BL and MN contributed with clinical expertise, validation of the analysis, and writing the manuscript. The content analysis defined two categories and five subcategories (Table 2).

**Table 2:** Overview of categories and subcategories

Categories	Subcategories
1. Different views of and ambivalence about the patient perspective in MDTMs	1.1 Medical versus holistic perspectives 1.2 Added value of the patient perspective 1.3 Possibilities for patient contributions
2. Prerequisites for the patient perspective being considered in MDTM decision-making	2.1 Structures promoting attention to the patient perspective 2.2 Determinants of registered nurses’ contributions

## Results

### Different views of and ambivalence about the patient perspective in MDTMs

All participants described the biomedical perspective as dominant in MDTMs. They were ambivalent about whether or not it was possible and/or desirable to include the patient perspective to obtain a more holistic basis for discussion and treatment recommendations.

#### Medical versus holistic perspectives

The MDTM was often described as a quality assurance process and as a conference where the biomedical perspective dominated. The MDTM was conducted to ensure the best treatment recommendations for each patient. However, participants’ views regarding what information should be included in MDTM case discussions differed, revealing various views about the purpose of the MDTM. Some participants argued that the MDTM case presentations and discussions should focus on medical information and that patient-related information should not influence MDTM recommendations. This position is exemplified in the following quotation from a participant:

*…as a patient, I need to be sure that the discussion about my illness is independent of where I come from, what I carry with me and what social network I have. (Focus group interview no. 1)*

In contrast, some participants considered the patient perspective central to ensuring a holistic approach to the MDTM case discussion and to providing individualized treatment recommendations. However, the question of including the patient perspective was understood to be complex, involving reflections on content, format, and responsibilities, and it was unclear how the perspective should be included and by whom it should be shared. To provide additional information and allow consideration of other aspects, some participants suggested a broader professional participation of, for example, physiotherapists, occupational therapists, dieticians, palliative care experts, and primary health care representatives. However, resource and time constraints were repeatedly described as barriers to include the patient perspective, leading some participants to question whether the involvement of additional health professionals was justified. In addition, ambivalence was expressed about sharing extensive patient perspective information because this would demand more resources and possibly limit the number of case discussions. Alternatively, more specialized MDTMs, for example, concerning rehabilitation or palliative care aspects, were suggested to facilitate more patient-centred approaches.

#### Added value of the patient perspective

Although the MDTM was described as a medical conference, several participants regarded the inclusion of the patient perspective as adding value to the discussion. References to patient perspective were broad, including social status and life situation, medical aspects such as physical status and comorbidity, and psychological and personal aspects such as patient preferences and views. According to the participants, such information was rarely included in MDTM discussions, and when it was, it was generally not decisive for the treatment recommendations. Some participants argued that the patient perspective was more important after the MDTM, as a foundation for how to care for the patient, than during the actual MDTM case discussions. One participant expressed this view as follows:

*…the psychosocial, it doesn’t change the medical assessment at the MDTM, // but it does affect how we handle the case [i.e., the patient]. (Focus group interview no. 1)*

The patient, together with the physician, was seen as responsible for deciding on treatment, and some participants stated that not taking the patient perspective into account would increase the risk that the final decision could deviate from MDTM recommendations. One participant explained this as follows:

*…the best possible medical decision is probably made at the MDTM by consensus. // …then it might be something completely different, in the end anyway, when the patient is told what the recommendation is. Then you weigh everything up in the meeting with the patient. And that’s where the decision is really made. (Focus group interview no. 2)*

This viewpoint had some participants arguing for the inclusion of patient perspectives in the MDTM case discussion to ensure that relevant treatment recommendations could be implemented.

#### Possibilities for patient contributions

Participants repeatedly stated that to include patient-related information in MDTM case discussions, patients needed to be able to contribute information relevant to them. Several participants claimed that most patients were informed that their case would be discussed at an MDTM in order to determine the best treatment option, but not all were alerted, which prevented them from contributing information. The importance of sharing information about treatment options and potential side effects in a way understandable to patients was described as a prerequisite for patient involvement. In this, the RN was seen as a mediator and interpreter of the information. According to some participants, patients often accepted MDTM treatment recommendations, but patients with low health literacy struggled to be involved in medical decisions. In such instances, patients should be able to rely on and trust health professionals to make correct judgments. As one participant stated it:

*…I often hear “we recommend that we do this”. In this way we [i.e., health care professionals] put a lot of responsibility on the patients to make the decisions for themselves. And I also find that there are many patients who are not able to make decisions, who don’t want to make the decisions, who want us to take all the responsibility. (Focus group interview no. 3)*

Some participants stated that patients and patient organizations demanded increased involvement in MDTMs. However, the general view was that the MDTM constitutes a professional forum in which patients, or patient representatives should not participate, since patient participation could introduce a risk of self-censorship and place the patient in an exposed situation. One participant stated:

*I think it would be very frightening for the patient in many ways. Also, I think it puts a filter on it [i.e., the case discussions]. Self-censorship comes in, because you…you have to choose your words very carefully. (Focus group interview no. 2)*

Patient involvement was seen in terms of the MDT considering the quality-of-life impacts of various treatment alternatives, and the patient getting the opportunity to contribute their perspective after the MDTM in discussing the recommendation.

### Prerequisites for the patient perspective being considered in MDTM decision-making

Participants repeatedly stated that the RNs’ possibilities to participate and their roles in the MDTMs varied greatly. Furthermore, given the different ways there were to collect information on the patient perspective, several participants suggested using standardized formats to gather such information.

#### Structures promoting attention to the patient perspective

Some participants considered assumptions about the patient perspective a barrier to the successful implementation of MDTM recommendations. Repeated reference was made to the lack of structured ways to collect patient-related information prior to MDTMs. For example, some participants reported not having contact with the patient prior to the MDTM, which made it difficult to collect relevant patient-related information. In such cases, information was gathered from colleagues and clinical files/referral texts rather than from the patients themselves. Such information was, however, often considered second-hand information not fully qualified for consideration as conveying the patient perspective in MDTMs. To ensure that attention was paid to the patient perspective, standardized and structured assessments of the patient perspective were proposed as a way to ensure that patients are given the possibility to express their views and opinions. Such developments could increase the quality of the information and strengthen the focus on the patient perspective in MDTMs. In contrast, other participants questioned the value of such assessments prior to the MDTMs because of the uncertain outcomes of case discussions. Instead, more flexible ways of working were proposed, allowing them to participate when it was specifically relevant to them, for example, in complex case discussions. There was recognition that the responsibility for collecting and presenting the patient perspective in MDTMs needed to be clarified. Some participants suggested that the RN and the physician share this responsibility. An alternate view was that it was not important who presented the information, but rather that the information was indeed available and discussed. One participant reflected on this point as follows:

*…why should you think that I as an RN am better at telling…what the patient*’s *perspective is than the physician (?) There is nothing that says it is. // The most important thing is that it’s told. (Focus group interview no. 4)*

#### Determinants of registered nurses’ contributions

RNs’ participation in and contributions to MDTMs were repeatedly described as hindered in three respects: barriers to attendance; perceived value of their information; and unclear role in the MDTM. Barriers to attendance consisted of RNs not always being considered mandatory participants in MDTMs and their participation being challenged by the timing of the MDTMs, which were planned around the physicians’ schedules. With MDTMs being time consuming and given the time allocated to their assignments, some participants said that they prioritized other work tasks over MDTM participation. Their contributions at the MDTMs was also hindered by the perceived value of their information. Some participants stated that their information on the patient perspective was generally not requested in MDTMs. This omission was concerning since the patient often shared relevant and personal information such as, for example, symptoms and worries with the RN. Several barriers to well-functioning teamwork in MDTMs were identified. One was hierarchical patterns that resulted in unbalanced participation with only a few active participants. Other factors were work experience, confidence, authority, discussion climate, and meeting structure. For some participants, contributing to the discussion was rather personal, requiring courage. One participant shared the following experience:

*Sometimes you have it on the tip of your tongue; however, as you said, both of us are thick skinned…but sometimes the skin disappears. (Focus group interview no. 3)*

Several participants described their roles in the MDTMs as unclear, with many disparate roles being reported, including coordinating investigations, planning, booking time slots, and contacting patients. Other participants primarily referred to a general educational aim. Despite ambiguous roles, the participants felt responsible for presenting the patient perspective at MDTMs and referred to themselves as patient advocates, spokespersons, or ambassadors. As an alternative to sharing the patient perspective in MDTMs, information was shared through documentation in medical records and during informal discussions with team colleagues. This experience was described by one participant as follows:

*…I understand that the medical perspective is enormously important. But sometimes the nursing perspective is just as important. And it feels a bit sad that it doesn*’t *come through. But we always find our own ways [to share information]//…you are the patient’s ambassador, in that way. (Focus group interview no. 2)*

Regardless of their role in the MDTMs, several participants talked of the benefit of receiving important information at the meetings, which increased their knowledge about the patients. Afterwards, when meeting with the patients, the RNs could repeat and explain the treatment recommendations from a nursing perspective and make sure that the patients based their decisions on the right information, and thereby support the patients in making informed decisions.

## Discussion

Aiming to explore RNs’ views of prerequisites for and barriers to inclusion of the patient perspective in MDTMs in Swedish cancer care, this study shows that there is ambivalence regarding the aim of MDTMs in terms of whether the MDTM should have a medical *or* a more holistic focus. This study also shows that although articulating the patient perspective in MDTMs was described as valuable, it was generally not found to be decisive for the treatment recommendations. Moreover, the study describes barriers to RNs’ participation in MDTMs and their sharing of information on the patient perspective.

Several studies emphasize the importance of including information on the patient perspective in MDTMs [15, 16, 33]. However, despite the term “patient perspective” being frequently used, there are various views of what the concept really means in clinical practice. In this study we adopted a comprehensive approach to the concept, allowing the participants to share their understandings. This resulted in broad descriptions that included social information, comorbidities, and patient views. Lack of a clear understanding of the patient perspectives concepts in the MDTM context has also been reported in studies from the UK [34], pointing to the need for a clear definition. Although the patient perspective was recognized as valuable, our results demonstrates that the RN’s experience, the aim of the MDTM, and the question if and in such case when the patient perspective should be considered is not fully explored. Since the MDTM, as a collective discussion and decision forum, aims to provide the best possible treatment recommendations for individual patients, the alignment of expectations in this area is needed to allow team members to agree on relevant information.

Lamb et al. [9] reported that patients should be represented by the MDTM participant who knows them best, which is concordant with this study showing that it is not important which profession presents the patient perspective, but does still suggest a shared responsibility between RNs and physicians. Bate et al. [35] also describe patients wanting their perspective to be represented by someone who has met them, but also often describing the RN as best suited for this task. According to other studies, this preference is partly because RNs have more continuity of patient contact throughout the clinical trajectory [5, 36], a finding in agreement with this study, in which the RNs described themselves as responsible to act on behalf of patients’ interests and to share information on the patient perspective. At the same time participants in our study describe their role during the MDTM as unclear, with the RN not always considered a mandatory team member. Participants often questioned the value of their contributions, since the information they could share was not asked for, an experience that possibly fed into the sense of an unclear role. Our results strengthen earlier studies that have reported limited or no contributions from non-medical disciplines in MDTMs [5, 16, 37], referring specifically to participants such as RNs. This lack of contribution can partly be explained by disease-focused case discussions and by MDTM discussions primarily driven by physicians [34]. It may also relate to the lack of defined roles in MDTMs in Swedish national guidelines. In addition, personal and team-related factors such as hierarchy, training in non-technical skills, work experience, confidence, and discussion climate all play roles, with an open climate as opposed to tension among participants possibly influencing RN contributions to case discussions [16, 38].

Our findings similarly suggest that participation in MDTMs was rather personal, requiring courage from the participants to contribute. Stewart et al. [39] reported that 19% of cancer nurse specialists (CNSs) who participated in MDTMs found the activity uncomfortable or intimidating, and only half of the respondents said that they would challenge MDTM participants. Other studies demonstrated that CNSs found alternative ways to contribute to MDTMs by sharing information, asking questions, using humour, providing practical alternatives, or by framing contributions in medical terms; these approaches affected the MDTMs in different ways by facilitating discussion, the decision-making process, and teamwork [8, 36]. An interesting ambivalence emerged in our study regarding participants reporting unclear roles in and limited contributions to MDTMs, while describing their responsibility to act on behalf of patients’ interests. This result leaves us with the following question: Do the RNs take a passive and subordinated position in the MDTM or do they value their contribution as insignificant? Simone Roach’s framework of caring, “six Cs”, include the attribute *commitment* [in caring], described as a complex affective response that influences the convergence between desires and obligations, including accepting limitations and prioritizing tasks [40]. This idea was reflected in this study by participants describing a high caseload at MDTMs and suggesting participating selectively in the cases relevant to them. This could potentially enhance commitment and contributions to the cases selected. Wallace et al. [8] supported this idea, reporting that RNs’ contributions to MDTMs were particularly relevant in complex and relapsed cases and when patient preferences, psychosocial factors, or communication difficulties could affect treatment decisions. Yet, this idea raises questions regarding who decides on the case selection criteria, when it is relevant to include patient-related information, and when RNs should participate. To support active contributions to MDTMs and an enhanced focus on the patient perspective, clarification of the national mission statement for CNs, covering the role description, and compulsory CN specialist course to support RNs’ active engagement in strengthening their role in MDTMs may be necessary. Work experience is suggested to impact on the RNs possibility to contribute to MDTMs [41] but whether RNs’ education level affect their contributions to the MDTMs is unknown and merits further investigation.

The participants’ ambivalence regarding the medical versus holistic perspectives and descriptions of an unclear role in MDTMs can be seen as symptoms of organizational ambiguity. If MDTMs are to be patient-centered, holistic information should be integrated in the discussion and cover medical factors as well as patient-related perspectives [8]. The results indicate that the patient perspective should preferably be shared by the patients themselves and not be collected from, for example, clinical files and referral texts, since these were considered insufficient. However, this study found that the opportunities to collect this information were hindered by a lack of structured assessment, unclear division of responsibilities within the MDT, and the fact that some RNs did not have contact with the patients before the MDTMs. In this study, participants proposed the use of structured assessments of the patient perspective to increase the value of the information but also to standardize information management. Taylor et al. [34] suggested that such developments would strengthen the RN’s role as an MDTM participant responsible for holistic needs. Introducing of such tools would require that the MDT define relevant patient-related aspects to be considered, since key information may vary between diagnoses and in relation to the treatment options discussed. Some participants in this study described a need to include other allied health professionals to uphold a holistic approach to the MDTM discussion, an approach that needs to be balanced against the effective use of resources. To address patients’ complex needs, Horlait et al. [5] argue for a culture change that enhances interdisciplinary teamwork. To achieve holistic discussions and individualized treatment recommendations, MDTs may need to develop interdisciplinarity rather than multidisciplinarity and integrate knowledge from several relevant sources and perspectives [5, 42]. This development would call for the clarification of roles and responsibilities, identifying factors that influence MDTM performance, team education, and structured team-based evaluations. As an alternative to extending the MDT to ensure a more holistic approach to the MDTM, some participants in our study suggested that the patient perspective rather be considered after the MDTM, potentially through establishing a separate forum to discuss the treatment recommendations from the patient’s perspective. This proposal is in line with a study by Winters et al. [43], which discussed alternative ways to integrate the patient perspective in MDTMs using “post-MDTM consultations” in which the biomedically based MDTM recommendations are adapted to the patients’ abilities, circumstances, and wishes.

To decrease potential organizational ambiguity, the aim of the MDTM may need to be more precisely defined, including a clearer description of what information should be discussed and the different participants’ roles and responsibilities. To determine whether the patient perspective should be included in MDTMs and how, the question of what benefits patients the most must be answered: Should their perspective be included in the MDTM discussion, or should the MDT conduct a medically focused discussion before considering the patient perspective [9]? In previous research, MDT members identified the specialist nurse as having an important role as the central point of contact for the patient, offering support throughout the clinical trajectory and advocating for the patient in the MDTM [44]. This is aligned with our results, in which RNs described themselves as responsible for presenting the patient perspective. Yet, in contrast, some participants stated that the MDTM case discussion should be medically focused and that RNs should only participate in selected case discussions. This challenges a patient-centered care approach and underlines the importance of clarifying the RN’s role and responsibilities within the MDTM.

## Strengths and limitations

This study explores a subject that has received limited attention in earlier research, namely, RNs’ views of the patient perspective in MDTMs. It provides a qualitative perspective and in-depth insights. Most of the included RNs participated regularly in MDTMs; a small number of RNs did not participate but were nevertheless well aware of MDTM procedures, bringing valuable information to the focus group interviews as they met the patients before and after MDTMs. A strength of this study is that the authors have various backgrounds and experiences in cancer care, with clinical, academic, and organisational experience ensuring a variety of perspectives. One author participated in a focus group interview before being recruited to the research group, which might be seen as introducing a potential bias [45, 46]. However, transparent and continuous discussions between the authors included self-reflection and self-scrutiny to ensure valid and grounded interpretations of the data [46]. Reliability was further strengthened by discussions leading to joint agreements on key themes and subthemes [27]. Credibility was strengthened by recurrent confirmation of the analysis with references to the transcripts and notes. The results are supported by representative quotations, increasing the conformability. A limitation of the study is that the participants reflected on their own experiences in the context of Swedish cancer care, making transferability less certain. Sample size is crucial for credibility [47] and our study is based on four focus group interviews with representation from different diagnostic areas, clinics, and hospital types. This is considered a strength since it enhance variation in the participating RNs’ knowledge of the research subject based on the varying roles of university and county hospitals, and on the fact that the RN role varies between diagnostic groups due to the complexity of the cancer diagnosis, the health care organization and the prerequisites for their assignments. Although we cannot exclude the possibility that including additional RNs could have brought additional perspectives, data saturation was nevertheless perceived to have been attained during the content analysis [47]. Furthermore, the inclusion of various hospitals and diagnostic areas may have blurred roles and responsibilities for the patient perspective in certain MDTs.

## Conclusions

This study reveals various views of information concerning consideration of the patient perspective in MDTMs and the need to clarify the RN’s role in these meetings. There was uncertainty surrounding the overall impact of the patient perspective, indicating an organizational ambiguity regarding MDTMs. As the patients do not participate in MDTMs, our results show that, if enhanced presentation of patient perspective in MDTMs is desired, key information points and structures must be established to collect and present relevant patient-related information. Also, further research is needed on how and when RNs should optimally be integrated throughout the MDTM decision-making process.

## Data Availability

The datasets generated and/or analysed during the current study cannot be shared publicly due to regulations in the Swedish Data Protection Act (2018:218; 2019:219) and Ethical Review Act (2003:460), but data are available from the corresponding author on reasonable request.

## Abbreviations

CN: Contact nurse
CNS: Cancer nurse specialists
MDT: Multidisciplinary team
MDTM: Multidisciplinary team meeting
RN: Registered nurse
